# A longitudinal neuroimaging study of adolescent girls’ mentalizing and perspective-taking tendencies

**DOI:** 10.1016/j.dcn.2025.101526

**Published:** 2025-02-07

**Authors:** Victoria Guazzelli Williamson, Marjolein E.A. Barendse, Samantha J. Chavez, John C. Flournoy, Theresa W. Cheng, Danielle Cosme, Michelle L. Byrne, Nicholas B. Allen, Jennifer H. Pfeifer

**Affiliations:** a Department of Psychology, University of Oregon, Eugene, OR, United States; b Department of Child and Adolescent Psychiatry/Psychology, Erasmus Medical Center, Rotterdam, Netherlands; c Department of Psychology, Harvard University, Cambridge, MA, United States; d Annenberg School for Communication, University of Pennsylvania, Philadelphia, PA, United States; e Turner Institute for Brain and Mental Health, School of Psychological Sciences, Monash University, Clayton, VIC, Australia

**Keywords:** Adolescence, Social cognition, Self-evaluation, Social neuroscience, Prosocial behavior, Social brain

## Abstract

Research in developmental psychology suggests that self-concept formation and mentalizing capacities, along with their neural foundations, show significant developmental change during adolescence. Perspective-taking tendencies are also believed to increase in adolescence, supporting the refinement of prosocial behavior and the demands of increasingly complex social relationships. To explore the development of, and relationship between, these processes in adolescence, early adolescent girls (N = 172) completed a measure of perspective-taking tendencies and a self-evaluation fMRI task at two waves, approximately 18 months apart (mean ages = 11.62 and 13.20, respectively). In line with our hypothesis, perspective-taking tendencies were positively associated with age. Greater perspective-taking tendencies were also associated with a more prosocial, and less antisocial, self-concept. In addition, dmPFC activity increased with age, but this did not survive correction for multiple comparisons across all mentalizing regions. Post hoc analyses also showed that an increase in perspective-taking tendencies across waves was significantly associated with activity in parts of the precuneus at wave 2. Finally, while we did not observe cross-variable coupling, our Bivariate Latent Change Score model showed that lower perspective-taking tendencies at wave 1 were associated with greater latent change in this variable (and the same was true for mean activity in mentalizing brain regions).

## 1 Introduction

Adolescence begins with the clear biological marker of puberty and culminates with the more ambiguous assumption of adult roles. Contingent on successful integration into society is the development of social skills, support networks, meaningful relationships, and an ability to navigate the social world around us. To succeed in these endeavors, we must develop social cognition—a term that encompasses the cognitive processes underlying our ability to understand social situations and people, including ourselves. Thus, it comes as no surprise that social cognitive development has been a longstanding area of focus in adolescent research. Adolescence is a period of identity formation and protracted development in our understanding of others (Crone and Fuligni, 2020, Pfeifer and Peake, 2012). During adolescence, we strive to develop a sense of ourselves that is clear and sophisticated (Branje, 2022, Erikson, 1968, Erikson, 1993; Klimstra et al., 2012; Van Der Cruijsen et al., 2023) while concurrently advancing our capacity to understand others (and ourselves) through the development of processes such as mentalizing and perspective-taking (Burnett et al., 2011, Desatnik et al., 2023, Dumontheil et al., 2010). These complementary and intimately intertwined processes—the development of the self and understanding of ourselves and others—support our ability to perform prosocial acts, develop strong connections with others, successfully navigate an increasingly complex social world, and, ultimately, find a meaningful place for ourselves within society (Crone et al., 2022, Crone and Fuligni, 2020, Fett et al., 2014, Hollarek and Lee, 2022).

### 1.1 Developmental changes in mentalizing during adolescence

Mentalizing is a broad social cognitive process involving considering individuals’ underlying mental states. Perspective-taking involves adopting the point of view of *others* (Guazzelli Williamson and Mills, 2023) while self-evaluation and self-processing involve thinking about one’s *self* (Pfeifer and Peake, 2012).

One classic measure of perspective-taking comes from the Perspective-Taking subscale of the Interpersonal Reactivity Index—a subscale that measures self-reported perspective-taking tendency (Davis, 1980). Despite conflicting cross-sectional findings (Hawk et al., 2013, Karniol et al., 1998, Yang and Kang, 2020), longitudinal studies and systematic reviews have found that self-reported tendencies to engage in perspective-taking (as measured via the IRI-PT) increase with age during adolescence (Davis and Franzoi, 1991, Hall et al., 2021, Van der Graaff et al., 2014). This suggests that during adolescence, young people increasingly believe they are more likely to consider the point of view of others.

Perspective-taking tendency has been associated with several functional outcomes, including social functioning, and self-esteem (Davis, 1983, De Corte et al., 2007). Perspective-taking has long been theorized to be related to prosocial behavior, including among adolescents, with some evidence that perspective-taking may even drive subsequent prosocial behavior (Eisenberg et al., 1991, Eisenberg and Fabes, 1990, Tamnes et al., 2018). On the other hand, while there is some evidence of a relationship between facets of empathy and antisociality, there is limited evidence that antisociality relates specifically to perspective-taking tendencies (Anastassiou-Hadjicharalambous and Warden, 2008, Anderson et al., 2024, Zafirakis, 2009). Additional longitudinal research among adolescents is needed to clarify the relationship between perspective-taking tendencies and prosociality and antisociality, as conflicting findings have been reported (Van der Graaff et al., 2018). Moreover, most of this work does not integrate or even account for self-concept—a key cognitive feature that is developing during adolescence. Research in this domain would thus benefit from integrating work on self-development in investigating self-perceived perspective-taking and adolescents’ perceptions of how prosocial and antisocial they are.

Adolescence is also a particularly important period for the development of the *social* self. Indeed, the most dramatic changes in self-concept development during adolescence are seen in social domains (Pfeifer et al., 2013, Pfeifer and Peake, 2012). For example, prior research has shown that while certain facets of self-esteem, such as appearance and athleticism, remain relatively stable, the social self undergoes significant, dynamic developmental changes during adolescence (Orth et al., 2021). These findings are further bolstered by neuroimaging evidence among early adolescents which has shown that activity in brain regions known to be involved in self–processing show stronger activity during social self-evaluations (e.g., as compared to non-social, academic self-evaluations; Pfeifer et al., 2013). Notably, trait self-evaluation has been shown to differ between boys and girls (Orth et al., 2021). While a meta-analyses found no gender differences in trajectories of trait self-evaluation, a longitudinal study focused specifically on social self-concept found steady decreases in adolescent girls’ social status in contrast to no change among boys (Orth et al., 2021, Rahal et al., 2020).

The majority of existing research relating adolescent perspective-taking and self-processing is cross-sectional and thus unable to parse developmental trajectories of these processes across adolescence. Thus, there is a gap in our knowledge of between- and within-person changes across the second decade of life. Larger longitudinal designs following the same individuals would afford the ability to track within-individual changes to help understand how these processes are developing while also allowing for the assessment of individual variability.

### 1.2 Development of brain regions involved in self-evaluation, mentalizing, and perspective-taking during adolescence

Self-evaluation is instantiated in brain activity and undergoes profound development during adolescence. Neural correlates of self-evaluation have consistently implicated cortical midline structures (CMS) (Northoff et al., 2006, Pfeifer and Berkman, 2018, Pfeifer and Peake, 2012, Qin and Northoff, 2011), including the medial prefrontal cortex (mPFC), anterior cingulate cortex (ACC), and medial posterior parietal cortex (including precuneus, posterior cingulate cortex, and retrosplenial cortex; Barendse, Cosme et al., 2020; Van der Cruijsen et al., 2019; Van Der Cruijsen et al., 2023). In addition, studies of self-evaluation among adolescents have consistently found activity in the bilateral temporoparietal junction (TPJ) and supramarginal gyrus (Barendse et al., 2020, Van der Cruijsen et al., 2019). The primary contrast of our self-evaluation task (described in Methods), revealed activity in the mPFC, ACC, precuneus, medial parietal cortex, and insula in our sample of 10–13-year-olds (Barendse et al., 2020).

Cognitive processes such as mentalizing and perspective-taking also reliably activate regions of the social brain that undergo structural and functional development during adolescence (Blakemore et al., 2007, Mills et al., 2014, Pfeifer et al., 2009, Van Den Bos et al., 2011). mPFC, TPJ, posterior superior temporal sulcus (pSTS), and anterior temporal cortex (ATC) are neural correlates of mentalizing that undergo significant developmental change across adolescence (Mills et al., 2014). Results from a meta-analysis suggest that activity in brain regions putatively associated with inferring and understanding the intentions of others (such as the anterior mPFC and TPJ) increase during adolescence (Crone and Dahl, 2012). Importantly, many of the regions involved in self- and other-understanding overlap. For example, while mPFC is a clear neural correlate of self-evaluation, it is also associated with mentalizing (Amodio and Frith, 2006) and first-person perspective-taking (Ames et al., 2008).

Empirical research suggests nuanced developmental trajectories of the function of brain regions involved in self-evaluation and mentalizing across adolescence. Some work has reported lower activity in mPFC generally, and dmPFC specifically, among older, compared to younger, adolescents during a variety of tasks involving mentalizing, reading about social emotions, thinking about intentions, or engaging in prosocial behavior (Blakemore et al., 2007, Burnett et al., 2009, Do et al., 2019, Gunther Moor et al., 2012). Thus, a number of theoretical accounts propose that mPFC activity, including dmPFC activity, peaks during early adolescence and/or decreases across adolescence, perhaps due to developmental shifts from less efficient social cognition and greater engagement in self-other integration by adolescents to greater efficiency and reliance on cognitive processes for understanding others in adulthood (Blakemore et al., 2007, Burnett et al., 2009, Crone and Fuligni, 2020, Grossmann, 2013, Johnson et al., 2009).

### 1.3 Implications of perspective-taking development for self-concept formation

The development of perspective-taking abilities and tendencies across adolescence has important implications for self-concept development. Some of the oldest and most influential theories of self-development suggest that self-concept development results, at least in part, from internalizing others’ beliefs about ourselves (Baldwin, 1895, Cooley, 1902, Mead, 1934). This requires taking another’s perspective of the self, otherwise known as engaging in a “reflected self-appraisal.” Understanding one’s identity in terms of social traits, in particular, may require incorporating reflected self-appraisals into one’s self-concept (Pfeifer et al., 2009). For instance, assessing one’s social traits, such as whether one is popular or loyal, may depend more on others’ appraisals rather than self-evaluation of physical traits. Of interest, during adolescence, we are concurrently refining our perspective-taking skills while developing a self-concept, which requires assessing where we fall on inherently social traits, such as whether we are cool. During this time, we are also especially sensitive to *social* stimuli such as peer- and social evaluations (Burnett et al., 2011, Steinberg, 2005). This increase in preoccupation with others’ opinions during adolescence may coincide with an increase in self-consciousness (Somerville, 2013). Thus, adolescence presents an important period to study these constructs and their potentially intertwined development.

Another example of the overlap in mentalizing and self-processing systems can be found in the neuroimaging literature on adolescent direct and reflected self-evaluations. Indeed, longitudinal work suggests that direct and reflected self-evaluation share common neural correlates (e.g., mPFC, dorsolateral PFC, precuneus, etc.) and show increasing overlap, both neurally and behaviorally, across adolescence (Van der Cruijsen et al., 2019). Direct self-evaluations are an individual’s own judgements about themselves (“I’m funny”), whereas reflected self-evaluations are evaluations of oneself through the perceived point of view of another (e.g., “My teacher thinks I’m smart” or “My mom thinks I’m irresponsible”). Compared to adults, adolescents have been shown to display increased engagement of mentalizing brain regions—including the dorsomedial prefrontal cortex (dmPFC) and temporoparietal junction (TPJ)—during direct self-evaluation. That is, even when asked to evaluate themselves from their own perspective, adolescents *spontaneously* activated regions of the brain involved in mentalizing and perspective-taking (Pfeifer et al., 2009). Other studies have corroborated these findings, providing cross-sectional evidence, for example, that during self-evaluations early adolescents elicit greater dmPFC activity than adults (Pfeifer et al., 2007). A recent longitudinal study of 10–24-year-olds found a quadratic relationship between age and mPFC activity for both direct and reflected self-evaluations, and a linear increase in right and left TPJ with age (Van Der Cruijsen et al., 2023). Overall, this work suggests that adolescents may activate brain regions involved in mentalizing and perspective-taking during direct self-evaluations—even when they are not explicitly instructed to perspective-take.

Few studies have probed individual differences and developmental changes in self-evaluation and mentalizing *together*, which is particularly important given the likely intertwined nature of these processes. In fact, perspective-taking and self-processing may be most connected in social self-evaluation compared to more individualized domains of self-concept like academics (Pfeifer et al., 2009). Given the behavioral and neural overlap in mentalizing and social self-evaluative processes, we sought to investigate whether activity in mentalizing regions during social self-evaluation is related to perspective-taking tendencies.

### 1.4 The present study

To fully understand the development of successful, prosocial adults with meaningful social connections, a clear sense of self, and an understanding of their place in society, we must understand the basic social cognitive processes that support these feats. While adolescence is an important period for the development of perspective-taking, mentalizing, and self-evaluation—all of which have been shown to differ between individuals—the nuanced individual differences and developmental trajectories of these processes are not sufficiently understood given a dearth of longitudinal research.

We use multilevel modeling of longitudinal data with two waves of IRI-PT scores and two waves of behavioral and neural activity during social self-evaluation among adolescent girls. This sample was drawn from an existing prospective longitudinal study of social and neural development of adolescent girls^1^ (Barendse, Vijayakumar, et al., 2020). We probe for individual differences and developmental changes in (i) perspective-taking tendencies and (ii) activity of mentalizing brain regions during social self-evaluation during early-to-middle adolescence. We also examine the relationship between perspective-taking tendency and activity in mentalizing brain regions during social self-evaluation.

Aims and hypotheses

The current study aimed to answer the following research questions during early-to-middle adolescence (aims and corresponding hypotheses were pre-registered on the Open Science Framework (OSF): https://osf.io/xmb9w/):

1. Do self-reported perspective-taking tendencies increase with age?

Hypothesis: We hypothesize that the tendency to take others’ perspectives (as assessed by IRI-PT scores) will be positively associated with age.

2. Does activation in mentalizing brain regions during social self-evaluation increase with age?

Hypothesis: We hypothesize that activation in mentalizing brain regions will be positively associated with age.

3. Are adolescents’ behavioral perspective-taking tendencies related to activity in mentalizing brain regions during social self-evaluation?

Hypothesis: We hypothesize that adolescents with greater perspective-taking tendencies (assessed by IRI-PT) will display greater concurrent activation in mentalizing brain regions during social self-evaluation.

4. Are latent changes in tendencies to take others’ perspectives related to latent changes in neural activation of mentalizing regions during social self-evaluation?

Hypothesis: We expect latent changes in tendencies to report taking others’ perspectives to correlate positively with latent changes in neural activation of mentalizing regions during social self-evaluation.

Additionally, we pre-registered an aim to model latent change of perspective-taking tendencies and mentalizing neural activity during social self-evaluation across both waves. Related methods and results (including planned contingency analyses i.e., change score linear regressions), can be found in supplementary materials.

## 2 Methods

### 2.1 Participants

The current study uses data from a larger longitudinal project, the Transitions in Adolescent Girls (TAG) study, which is described in detail in a previous publication (Barendse, Vijayakumar, et al., 2020). Methods reported herein focus on data collected from the first and second waves of the project. 174 adolescent girls^1^ aged 10.00–12.99 years (*M*=11.64) participated in a longitudinal study of social and neural development. Girls were recruited from the community, together with one of their parents/guardians. Inclusion criteria included: 10.00–12.99 years old at time of enrollment, fluent in English; and having normal or corrected-to-normal vision. Exclusion criteria included taking psychotropic medication at time of enrollment; MRI contraindications including claustrophobia and presence of ferromagnetic material in the body); reporting or suspecting being pregnant; and diagnoses of a developmental disability, psychotic disorder, or behavioral disorder (including autism). Families were recruited primarily through recruitment letters distributed by schools to families with children registered as female students in the greater Eugene/Springfield area (Lane County, Oregon, USA), and to a minimal extent from secure databases of people who registered their interest in our lab’s/department’s research, recruitment flyers posted around the community or disseminated at community events, and through snow-balling efforts. Parents/guardians gave informed consent and adolescents gave assent to participate. Ethics approval was received from the Institutional Review Board of the University of Oregon.

At wave 1, participants came in for two sessions and completed a series of self-report questionnaires, including the perspective-taking subscale of the IRI (Davis, 1980) at session 1, at home, or at session 2. At session 2 they completed an MRI session including structural, diffusion, resting-state functional and task-based functional scans. Approximately 18 months later (*M* = 1.57 years, *SD* = 0.12 years), 163 girls aged 11.08–14.50 years (*M* = 12.75) completed wave 2, which again included two sessions and the same assessments described for wave 1. At wave 2, all participants completed the IRI-PT at home between sessions 1 and 2, or at session 2 if they had not finished it by then.

### 2.2 Interpersonal reactivity index-perspective-taking (IRI-PT) subscale

The IRI-PT subscale is a cognitive measure of “the tendency to spontaneously adopt the point of view of others” (Davis, 1980). Response options for each question from the 7-item subscale were a 5-point Likert scale ranging from 0 ("does not describe me well") to 4 ("describes me very well"). The subscale is considered a measure of cognitive empathy, as opposed to affective interpersonal reactivity (e.g., empathic concern; see Supplementary Figure S2 for divergence in IRI subscales). The IRI-PT has good internal consistency in our sample and others (Cronbach’s alpha of 0.75 among males and 0.78 among females in Davis (1980); and standardized Cronbach’s alpha of 0.67 at wave 1 and 0.78 at wave 2 among girls in our sample).

Individuals who answered at least 5 of the 7 IRI-PT items were included in the analysis. If participants only answered 5 or 6 of the 7 IRI-PT items, a total IRI-PT score was calculated by generating any missing scores based on their average score from the questions that they did answer. We pre-registered that data would be transformed to obtain an approximate normal distribution of the distribution if IRI-PT scores were skewed. As data were relatively normally distributed (skewness_wave1_: −0.660; kurtosis_wave1_: 1.406; skewness_wave2_: −0.045; kurtosis_wave2_: −0.292) we did not transform IRI-PT scores (Supplement Fig. 1). Outliers on the IRI-PT were winsorized to 1 % above the next highest or below the next lowest value (N_wave1_ = 2; N_wave2_ = 0). Following winsorization, IRI-PT scores for skewness and kurtosis at wave 1 were −0.276 and −0.010, respectively. Following cleaning, 126 IRI-PT scores were available at wave 1 and 135 were available at wave 2 (see Table 1 for description of raw IRI-PT scores by wave and Supplementary Figure S1 for distribution of IRI-PT scores before and after winsorization).

**Fig. 1:**
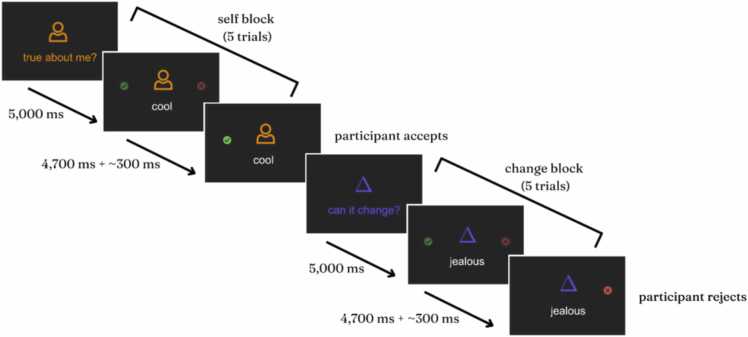
Self Versus Change Task. Fig. 1 Legend. Schematic of Self Versus Change fMRI Task. This figure shows the design of the Self Versus Change task. Each run consists of ten trials: five trials where participants respond about whether a trait adjective describes (“self” block) them followed by five trials where participants respond about whether a trait adjective can change among people in general (“change” block).

**Table 1:** Self versus change fMRI task trait adjectives.

Legend. This table lists all Self Versus Change fMRI Task Adjectives. Adjectives belong to one of three conditions: i) prosocial, ii) social status, or iii) antisocial. Positively-valenced adjectives are depicted in blue while negatively-valenced adjectives are depicted in red.

### 2.3 fMRI social self-evaluation task

The self-evaluation fMRI paradigm is based on our previous research on self-evaluation in adolescents (Cosme et al., 2019, Jankowski et al., 2014, Pfeifer et al., 2007, Pfeifer et al., 2013). In this social self-evaluation paradigm, adolescents were presented with 50 individual social trait adjectives that are relevant to interpersonal relationships (e.g., friendly, respectful, awkward, selfish, popular, aggressive). Exploratory factor analysis in a pilot sample of 100 late adolescent females identified an optimal three-factor solution for all trait adjectives, which we refer to as falling under the following three categories: prosocial, antisocial, and social status (see Table 1 for a full list of items and Barendse, Vijayakumar, et al. (2020) for additional information).

Participants were instructed to make a binary choice response at every trial. In the social self-evaluation “self” condition, participants reported whether a given trait describes them, and in the “change” condition participants reported whether they believe the trait is something that can change about people in general (i.e., is malleable). Building on previous work, this task is designed to isolate self-processing (Jankowski et al., 2014; Cosme et al., 2022). Thus, participants are asked to think about the same traits in the condition of interest (“self”) and the control condition (“change”), such that the primary difference between the conditions is whether they are thinking about the adjectives in relation to themselves. Every trait adjective was presented for 4.7 seconds. Participants could respond at any time by pressing a button on a button box and reaction times were recorded. The task used a mixed event-related design with alternating “self” and “change” blocks of five traits (see Fig. 1 for task schematic). The task was split into two runs; traits that were presented in the “change” condition in run 1 were presented in the self-evaluation condition in run 2 and vice versa. The order of traits was randomized (Barendse, Cosme, et al., 2020).

### 2.4 fMRI data acquisition and preprocessing

Participants completed a mock scan prior to the MRI scan to familiarize them with the scanner. They received instructions for the task and practiced the task during the mock scan. Acquisition parameters for the T2*-weighted functional runs were as follows: 2 × 180 volumes of 72 slices with 2 mm isometric voxels, TR = 2000 ms, TE = 25 ms, multiband acceleration factor = 3, in plane acceleration factor = 2, FOV = 208 mm, flip angle = 90°, duration = 6.5 min per run. T1-weighted images were acquired as follows for co-registration: sagittal 3D MP-RAGE, 176 slices with 1 mm isometric voxels, FOV = 256 mm, TR = 2500 ms, TE = 3.41 ms, flip angle = 7°, TI = 1100 ms, matrix size = 256 × 256, acceleration factor = 2.

fMRIPrep version 1.5.2 was used to preprocess the fMRI data (https://fmriprep.readthedocs.io/en/stable/; (Esteban et al., 2019). The processing of the T1-weighted images included correction for intensity non-uniformity using N4BiasFieldCorrection (v2.2.0) and skull-stripping using AntsBrainExtraction (v2.2.0). Brain surfaces were reconstructed from the T1-weighted volumes using recon-all (FreeSurfer v6.0.1) and this was used to refine the brain mask estimated in the previous step. Brain tissue segmentation of cerebrospinal fluid (CSF), white-matter (WM) and gray-matter (GM) was performed on the brain-extracted T1-weighted images with FSL’s FAST (FSL v5.0.9). Finally, spatial normalization to MNI-space (ICBM 152 Nonlinear Asymmetrical template version 2009c) was performed through nonlinear registration with the antsRegistration tool of ANTs v2.2.0, using brain-extracted versions of both T1w volume and template.

Functional data were motion-corrected with MCFLIRT (FSL v5.0.9). Distortion correction was performed using field maps processed with FUGUE (FSL v5.0.9). If field maps were not available (n = 17), fieldmapless distortion correction was performed. Next, co-registration to the corresponding T1-weighted image was performed using boundary-based registration with six degrees of freedom (bbregister in FreeSurfer v6.0.1). Motion correcting transformations, field distortion correcting warp, BOLD-to-T1w transformation and T1w-to-MNI template warp were concatenated and applied in a single step using antsApplyTransforms (ANTs v2.2.0) with Lanczos interpolation. Finally, preprocessed functional data were smoothed using a 6 mm full-width at half maximum (FWHM) smoothing kernel.

The motion-related output from fMRIPrep was fed into an automated motion classifier (auto-motion-fmriprep, https://github.com/dcosme/auto-motion-fmriprep; Cosme et al., 2018). This tool is a trained classifier that utilizes the motion confound files generated by fMRIprep and classifies whether or not fMRI volumes contain motion artifacts. For neuroimaging analyses, participants with incomplete fMRI data (no scan or less than one run) were excluded (N_wave 1_ = 20, N_wave 2_ = 35). Participants whose scans contained motion artifacts in > 20 % of volumes overall based on the automated classifier were excluded (N_wave 1_ = 11, N_wave 2_ = 3). Additional participants were excluded for artifacts related to ceramic braces (N_wave 1_ = 0, N_wave 2_ = 2).

Subject-level models were set up and estimated in Statistical Parametric Mapping 12 (SPM12). For scripts, see https://github.com/dsnlab/TAG_scripts. For each subject, event-related models were estimated using a general linear model, a canonical hemodynamic response function, high-pass filtering of 100 seconds, and a first-order autoregressive error structure. Separate regressors for the “self” and “change” conditions and events were modeled with reaction time set as the duration. Trials with no response were modeled in a separate condition of no interest with duration per trial set at 4.7 seconds. Motion parameters (Euclidean distance, Euclidean rotation, the first derivatives of both, and the binary regressor from the automated motion classifier described above) were included as regressors of no interest. Linear contrasts for self > change were estimated for each subject.

### 2.5 Creation of regions of interest (ROIs)

Neural Regions of Interest (ROIs) were calculated using the “Mentalizing: Association Test” layer (https://www.neurosynth.org/analyses/terms/mentalizing) from Neurosynth, an automated meta-analysis tool (Yarkoni et al., 2011). Association test refers to “z-scores from a two-way ANOVA testing for the presence of a non-zero association between term use and voxel activation” (Neurosynth: FAQs, n.d.). The mentalizing meta-analysis version used was based on 151 studies and was downloaded on April 19th, 2020. Using FMRIB Software Library (FSL version 6), ROIs were obtained by binarizing voxels at a z-score of 5. We chose a high z-value to be conservative and only included areas consistently activated during mentalizing tasks. Additionally, we selected clusters with more than 66 contiguous 2 mm isotropic voxels as this aligned with our previous work determining a cluster-forming threshold for cluster family-wise error (FWE) correction in the same dataset (Barendse, Cosme, et al., 2020). While ROIs were derived from meta-analytic data instead of our task, a threshold of 66 contiguous voxels was used (which matches the extent threshold identified in our own task data) because we were mapping these ROIs onto task data and were not interested in very small structures. This yielded 10 clusters. Three clusters in the cerebellum and two clusters in the anterior temporal cortex were removed as they are not reliably activated in higher-order mentalizing and self-evaluative processes such as reflected self-appraisals (D’Argembeau et al., 2007; Ochsner et al., 2005; Pfeifer et al., 2009). Thus, we were left with 5 ROIs: the dmPFC, vmPFC, rTPJ, lTPJ, and precuneus. The thresholded Neurosynth ROIs used in these analyses are publicly available as NIfTI files on our Open Science Framework repository at https://osf.io/xmb9w/. Mean activation in each ROI was extracted for each participant and wave using 3dmaskave in Analysis of Functional NeuroImages (AFNI_18.2.04).

### 2.6 Statistical analyses

Hypothesis 1 stated that the tendency to take others’ perspectives (as assessed by IRI-PT scores) would be positively associated with age. This was tested using linear mixed-effects models implemented in the lme4 package in R version 4.3.1 (R Core Team, 2023). Age was modeled as a fixed effect with IRI-PT scores as the outcome. The intercept was allowed to vary randomly across participants, leading to the following equation: IRI-PT_ij_ = g_00_ + g_01_ * age + u_0j_ + e_ij_ (lmer syntax: IRI-PT ∼ age + (1 | ID)). To avoid overfitting, random slopes were not included in the model. Confidence intervals (95 % CI) and standardized estimates reported in text were estimated using the standardize_parameters function, employing the refit method for standardization. The association was considered statistically significant at an alpha-level of 0.05.

### 2.7 Hypotheses 2 & 3

For Hypothesis 2 and Hypothesis 3, we focused on relating age and IRI-PT scores, respectively, to univariate activity in particular ROIs in the self > change contrast.

ROI analyses testing Hypotheses 2 and 3 used linear mixed-effects models with lme4 in R. ROI activity was the outcome variable and the intercept was allowed to vary randomly across participants. To avoid overfitting, random slopes were not included in the model. For Hypothesis 2, age was modeled as a fixed effect. The statistical equation for Hypothesis 2 was as follows: ROI ∼ age + (1 | ID) and in mathematical notation: ROI_ij_ = g_00_ + g_01_ * age + u_0j_ + e_ij_. For Hypothesis 3, IRI-PT was modeled as a fixed effect. The statistical equation for Hypothesis 3 was as follows: ROI ∼ IRI-PT + (1 | ID) or in mathematical notation: ROI_ij_ = g_00_ + g_10_ + IRI-PT + u_0j_ + e_ij_. Each ROI was modeled separately and we corrected for multiple comparisons across the 5 ROIs using Benjamini-Hochberg correction (Benjamini & Hochberg, 1995).

Hypothesis 4 For hypothesis 4, we conducted a bivariate latent change score analysis using IRI-PT and ROI activation data from waves 1 and 2 (as specified in Kievit et al., 2018). The equation for hypothesis 4 was as follows: *ΔIRI-PT1 ∼∼ ΔROI1*. This analysis was conducted in R using the Lavaan package. Full Information Maximum Likelihood (FIML) was used to handle missing data. Model fit was prespecified to be judged based on the following fit indices: chi-square, Comparative Fit Index (CFI > 0.90), Standardized Root Mean Square Residual (SRMR < 0.08), and Root Mean Square of Error Approximation (RMSEA < 0.08) (Hu and Bentler, 1999, Schermelleh-Engel et al., 2003). We determined adequate model fit if at least two of the three fit indices met these criteria.

We pre-registered an aim to model mentalizing ROIs as a latent variable which was then input into a bivariate latent change score (BLCS) model along with IRI-PT scores. Results from our Confirmatory Factor Analysis (CFA) of the latent mentalizing neural variable and the BLCS with the latent mentalizing neural variable raised concerns (see Supplementary Materials). Thus, we report in text findings from the same model with mean ROI activity, as opposed to a latent variable of ROI activity.

### 2.8 Post hoc analyses: relating IRI-PT and pre-registered ROIs to trait adjective endorsement

We also conducted a post hoc analysis relating both IRI-PT and ROI activation to responses to the three trait adjective types from the social self-evaluation fMRI task: prosocial, social status, and antisocial trait adjectives. We pre-registered our hypothesis that IRI-PT scores would be positively associated with prosocial trait adjective endorsement and negatively associated with antisocial trait adjective endorsement. For each participant at each wave, a proportion was computed indicating their endorsement of each adjective type. For instance, the proportion of prosocial adjectives endorsed was computed by dividing prosocial adjectives endorsed by the total number of prosocial adjectives the participant responded to. Similarly, the proportion of antisocial adjectives endorsed was calculated by dividing the total number of antisocial adjectives they endorsed by the total number of antisocial adjectives they responded to. Social status adjectives included both positively- and negatively-valenced traits and thus proportion of endorsements of social status adjectives were calculated by summing positive social status adjectives endorsed and negative social status adjectives rejected, and dividing this number by the total number of social status adjectives responded to. Similar to Hypotheses 1–3, these models were tested using linear mixed-effects models implemented in the lme4 package in R version 4.3.1 (R Core Team, 2023). The proportion of adjectives endorsed for a given adjective type was the outcome, with IRI-PT or ROI activation (depending on the model) serving as the fixed effect.

### 2.9 Post hoc analyses: relating IRI-PT and pre-registered ROIs to trait adjective endorsement

We also conducted a series of exploratory post hoc analyses to complement our a priori, pre-registered methods. Specifically, we ran whole-brain voxel-wise analyses in SPM12 to identify any other brain regions in the “self” compared to “change” condition, as well as in each of the “self” and “change” conditions compared to baseline, that were concurrently associated with IRI-PT scores or related to change in IRI-PT scores across waves. We used the joint magnitude-extent threshold of 0.001 uncorrected, 66 voxels to be consistent with our prior work in this sample using these fMRI data (Barendse, Cosme, et al., 2020). (Table 2)

**Table 2:** Sample descriptive and demographic information.

	Wave 1 (rounded % of wave 1 sample with IRI-PT *or* useable task fMRI data)	Wave 2 (rounded % of wave 2 sample with IRI-PT *or* useable task fMRI data)
IRI-PT total scores or useable task fMRI data at given wave	*N* = 164	*N* = 157
Age	Mean (*SD*) = 11.62 (0.82); Range = 10.02–13.16	Mean (*SD*) = 13.20 (0.84); Range = 11.52–15.00
IRI-PT Total Score	*N* = 126 (77 %); Mean (*SD*) = 15.69 (4.00); Range = 0–24	*N* = 135 (86 %); Mean (*SD*) = 16.47 (3.99); Range = 7–26
Hispanic/Latino	*N* = 20 (12 %)	*N* = 20 (13 %)
Non-Hispanic/Latino	*N* = 140 (85 %)	*N* = 133 (85 %)
Asian	*N* = 10 (6 %)	*N* = 8 (5 %)
Black/African American	*N* = 9 (5 %)	*N* = 11 (5 %)
Native Hawaiian or Pacific Islander	*N* = 1 (1 %)	*N* = 1 (1 %)
Native American or Native Alaskan	*N* = 12 (7 %)	*N* = 8 (5 %)
White	*N* = 148 (90 %)	*N* = 139 (89 %)
Other	*N* = 11 (7 %)	*N* = 9 (6 %)
Multiracial	*N* = 27 (16 %)	*N* = 19 (12 %)

Legend: Note that *N*s are calculated based on individual participant endorsement of a given race/ethnicity at each wave. Data are reported at each wave such that if a participant had usable Self Versus Change fMRI data *or* IRI-PT data at a given wave, they were included in the race/ethnicity reports for that respective wave. Individuals may have selected more than one racial/ethnic identity (i.e., a participant who identifies as Black/African American, White, and Hispanic/Latino). *N*s for the multi-racial category were calculated based on individuals who endorsed more than one of the following: Asian; Black/African American; Native Hawaiian or Pacific Islander; Native American or Native Alaskan; White; or Other. Note that some participants entered their nationality in the Other or Multiracial categories and that these individuals are included in the “Other” and “Multiracial” counts.

## 3 Results

### 3.1 Endorsement of self-concept trait adjectives

The mean proportion endorsed for prosocial adjectives was 93.32 % and 93.46 % at waves 1 and 2, respectively. Positive social status trait endorsement (proportion of positively valenced social status traits endorsed plus negative social status traits rejected, divided by total social status traits responded to) was 68.17 % and 64.58 % at waves 1 and 2 respectively. The proportion of antisocial adjectives endorsed was 20.37 % at wave 1 and 24.68 % at wave 2.

Hypothesis 1 Relationship Between Age and Self-Reported Perspective-Taking Tendency

**Fig. 2:**
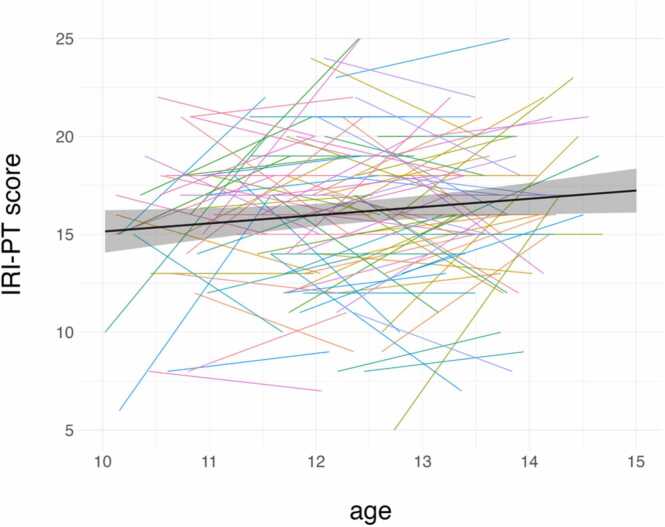
Association between age & IRI-PT score. Fig. 2 Legend: The black line represents the predicted values based on the mixed-effects model, and depicts a positive relationship between age and IRI-PT scores. The colored lines represent raw (not model-predicted) individual trajectories. The 95 % confidence interval is depicted via a gray band.

As expected, self-reported perspective-taking tendencies (measured via IRI-PT scores) were positively associated with age (β = 0.12, 95 % CI = (0.01, 0.23), *SE* = 0.19, *t* = 2.15, *p* = .033, *df* = 222.74) but included substantial individual variability (*SD* of random effect = 2.66). Average and individual trajectories are visualized in Fig. 2.

Hypothesis 2 Relationship Between Age and Activity in Mentalizing ROIs

**Fig. 3:**
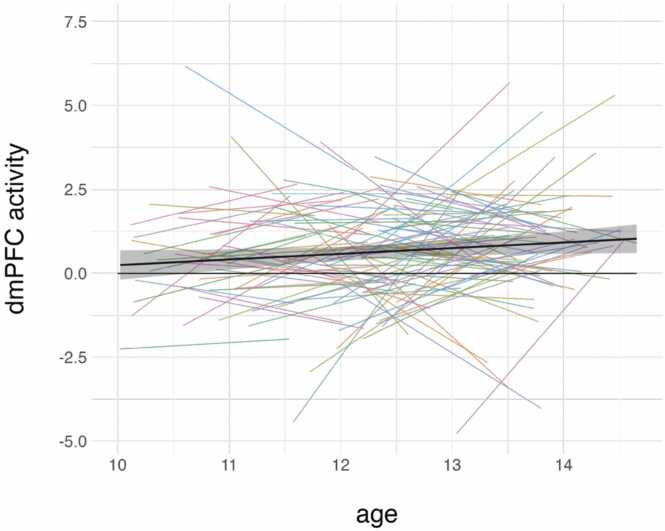
Association between age and activity in dorsal medial prefrontal cortex (dmPFC). Fig. 3 Legend: The black line represents the predicted values based on the mixed-effects model, whereas the colored lines represent raw (not model-predicted) individual trajectories. The 95 % confidence interval is depicted via a gray band. A second black line is depicted at the zero line to improve readability and show that the task reliably activates this region during self > change after about age 11.5 years.

The relationship between age and activity in each mentalizing ROIs and age were not significant after correction for multiple comparisons (Fig. 3; Table 3; Figure S4). While mixed-effects models revealed a positive relationship between age and activity in the dmPFC (β = 0.12, 95 % CI = (0.00, 0.24), *SE* = 0.08, *t* = 2.01, *p* = .045; *SD* = 0.57; *df* = 273.41), this relationship was no longer statistically significant after conducting pre-registered Benjamini-Hochberg correction for multiple comparisons of the five ROIs. Fig. 3 and Supplementary Figure S4 depict variability at the individual level in the relationship between individual ROIs and age.

Hypothesis 3 Relationship Between Self-Reported Perspective-Taking Tendencies and Activity in Mentalizing ROIs During Social Self-Evaluation

**Table 3:** Relationship between age and activity in mentalizing ROIs.

ROI ∼ age	β (95 % CI)	*SE*	*df*	*t*	*p*	*SD* of random effect
dmPFC ∼ age	0.12 (0.00, 0.24)	0.08	273.41	2.01	.045	0.57
vmPFC ∼ age	0.02 (−0.10, 0.14)	0.10	273.99	0.29	.770	0.54
rTPJ ∼ age	−0.08 (−0.20, 0.04)	0.06	273.44	−1.39	.167	0.23
lTPJ ∼ age	−0.08 (−0.20, 0.04)	0.07	272.36	−1.34	.181	0.50
precuneus ∼ age	0.07 (−0.05, 0.18)	0.07	273.30	1.10	.273	0.51

Legend: Table 3 shows statistics from the model testing Hypothesis 2 (ROI_ij_ = g_00_ + g_10_ + age + u_0j_ + e_ij_) including standardized effect size (β), 95 % confidence intervals (95 % CI) of the standardized effect size, standard error (*SE*), degrees of freedom (*df*), *t*-values, *p*-values, and the standard deviation (*SD*) of the random effect. Standardized estimates and 95 % CIs were estimated using the standardize_parameters function, employing the refit method for standardization. P-values shown here are from the model output and not corrected for multiple comparisons.

**Fig. 4:**
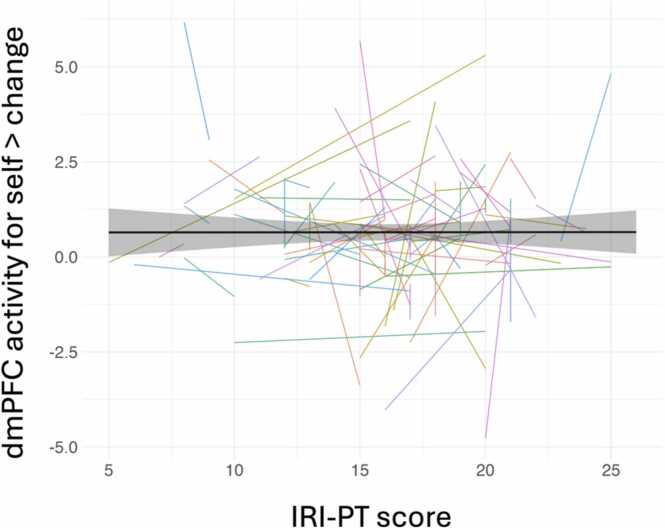
Association between IRI-PT score and activity in dorsal medial prefrontal cortex (dmPFC). Fig. 4 Legend. The black line represents the predicted values based on the mixed-effects model testing Hypothesis 3, whereas the colored lines represent raw individual trajectories. The 95 % confidence interval is depicted via a gray band.

Plots depicting the relationship between IRI-PT scores and activity in each of the ROIs can be seen in Fig. 4 and Supplementary Figure S5. Here as well, individual variability in this relationship is evident and there is no clear general trend at the group level. That is, IRI-PT scores are not strongly associated with activity in any of these individual regions (Table 4; Fig. 4; Supplementary Figure S5). In line with our pre-registration addendum and to account for the age-related variability we found in IRI-PT scores (Hypothesis 1), we also ran multilevel models with age as a covariate and found no statistically significant relationships between IRI-PT and each ROI. Note that the multilevel model relating IRI-PT scores and vmPFC activity generated a singular fit warning and a low standard deviation of the random effect, suggesting that there is not significant individual variability in vmPFC activity after accounting for IRI-PT scores, or that this variance is so low that it is difficult to estimate. As a robustness check, we also ran a Bayesian multilevel model with uninformative priors using ‘brms’ set with four chains, a 2000 iteration count, a warm-up phase of 1000 iterations, and adjusted with an adapt_delta of 0.99 and maximum tree depth of 15 (Bürkner, 2017). Main effects for the Bayesian model did not meaningfully differ (i.e., the β relating vmPFC and IRI-PT was −0.00), although the SD of the random effect was higher at 0.36, suggesting that the Bayesian model may more accurately capture individual variability in vmPFC activation after controlling for IRI-PT scores.

Hypothesis 4 Using a Bivariate Latent Change Score Model Relating ROI Activation and Perspective-Taking Tendencies to Test for Correlated Latent Change

**Table 4:** Association between IRI-PT score and activity in mentalizing ROIs.

ROI ∼ IRI-PT	β (95 % CI)	*SE*	*df*	*t*	*p*	*SD* of random effect
dmPFC ∼ IRI-PT	0.00 (−0.14, 0.14)	0.03	188.68	0.02	.987	0.40
vmPFC ∼ IRI-PT	−0.01 (−0.14, 0.13)	0.03	214.00	−0.12	.908	0.00
rTPJ ∼ IRI-PT	0.07 (−0.06, 0.21)	0.02	184.04	1.06	.292	0.34
lTPJ ∼ IRI-PT	0.09 (−0.04, 0.23)	0.02	187.87	1.35	.178	0.48
precuneus ∼ IRI-PT	0.11 (−0.03, 0.24)	0.03	195.05	1.53	.128	0.57

Legend. Table 4 shows statistics from the model testing Hypothesis 3 (ROI_ij_ = g_00_ + g_10_ + IRI-PT + u_0j_ + e_ij_) including standardized effect size (β), 95 % confidence intervals (95 % CI) of the standardized effect size, standard error (*SE*), *t*-values, *p*-values, and the standard deviation (*SD*) of the random effect. Standardized estimates and 95 % CIs were estimated using the standardize_parameters function, employing the refit method for standardization. *p*-values shown here are from the model output and not corrected for multiple comparisons.

**Fig. 5:**
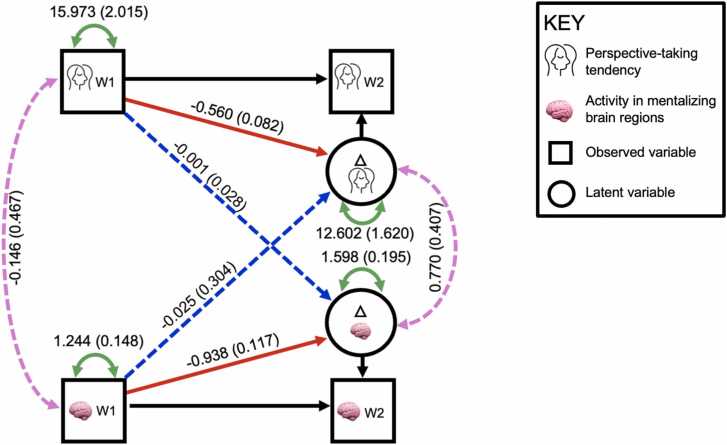
Bivariate Latent Change Score Model Relating IRI-PT and Mean Activity in Mentalizing ROIs During Social Self-Evaluation. Fig. 5 Legend. This BLCS model shows significant paths indicated via solid lines and non-significant paths represented via dashed lines. Estimates for each path are presented, with standard errors listed in parentheses. Latent variables are indicated via circles (e.g., latent change scores) whereas observed variables are depicted via squares.

Here we report a BLCS model run with mean ROI activation across mentalizing brain regions during self > change for the observed neural variables at waves 1 and 2 (Fig. 5). This model converged after 78 iterations and additional model output is available on OSF (https://osf.io/xmb9w/). As the model was just-identified, fit indices are not reported. Our hypothesis that latent change in perspective-taking tendencies and mentalizing ROI activation would be correlated was not supported. In addition, cross-variable paths were not significant (e.g., IRI-PT scores did not predict latent change in mentalizing ROI activity and vice versa). However, greater IRI-PT scores at the first wave significantly predicted decreased latent change in IRI-PT scores across waves. Similarly, greater activity in the mentalizing neural variable at wave 1 significantly predicted decreased latent change in the mentalizing neural variable across the two waves.

### 3.2 Endorsement of trait adjectives in relation to perspective-taking tendencies and activation in mentalizing ROIs

We also conducted a post hoc analysis relating IRI-PT and ROI activation to responses from the social self-evaluation fMRI task. In line with our pre-registered hypotheses, higher IRI-PT scores were positively associated with percentage endorsement of prosocial adjectives (i.e., self-evaluated prosociality) and negatively associated with percentage endorsement of antisocial adjectives (i.e., self-evaluated antisociality; see Table 5). As expected, self-evaluated prosociality and antisociality were moderately negatively correlated (-0.38 at wave 1 and −0.41 at wave 2). However, social status and antisociality were not meaningfully correlated, and the correlation between social status and prosociality increased from wave 1 (0.14) to wave 2 (0.32; see correlation matrix in Supplementary Figure S2). IRI-PT scores were not significantly related to social status adjective endorsement (calculated via positive adjectives endorsed + negative adjectives rejected, divided by the total number of adjectives with a response). Following reviewer feedback, we broke up the social status trait endorsement by valence, resulting in two proportions: (1) positive social traits endorsed divided by positive social status traits responded to and (2) negative social traits endorsed divided by negative social status traits responded to. Separating social status traits by valence did not make a difference in our findings as none of the models relating positively-valenced or negatively-valenced social status proportions to IRI-PT or ROI activity were significant. All models relating individual and mean ROI activity to self-reported social self-evaluation data from the task were not significant (Supplementary Tables S1 - S6). Findings did not differ when re-running the models controlling for age.

**Table 5:** Multilevel models associating endorsement of each adjective type to IRI-PT scores.

	β (95 % CI)	*SE*	*df*	*t*	*p*	*SD* of random effect
Prosocial ∼ IRI-PT	0.22 (0.09, 0.34)	0.03	239.47	3.52	.001	0.07
Social Status ∼ IRI-PT	−0.01 (−0.13, 0.12)	0.00	237.98	−0.13	.896	0.16
Antisocial ∼ IRI-PT	−0.27 (−0.39, −0.15)	0.00	240.80	−4.34	.000	0.12

Legend. Table 5 shows statistics from the model testing our post hoc hypotheses that IRI-PT scores would be positively associated with self-evaluated prosociality and negatively associated with self-evaluated antisociality (Trait_ij_ = g_00_ + g_10_ + IRI-PT + u_0j_ + e_ij_). Statistics reported include standardized effect size (β), 95 % confidence intervals (95 % CI) of the standardized effect size, standard error (*SE*), *t*-values, *p*-values, and the standard deviation (*SD*) of the random effect. Standardized estimates and 95 % CIs were estimated using the standardize_parameters function, employing the refit method for standardization. P-values shown here are from the model output and not corrected for multiple comparisons.

### 3.3 Exploratory whole brain analyses

We conducted a series of exploratory post hoc whole-brain voxel-wise analyses to search for any regions that may have not been identified using our a priori, pre-registered methods. While most of these analyses produced no significant results, all output from the models is reported in Supplementary Table S7. At the second wave, there was a region of the precuneus that was related to change over time in IRI-PT scores, suggesting that individuals with a greater increase in perspective-taking tendency across the waves displayed higher activity in this region at wave 2 in the “self” condition compared to the “change” condition. This region was adjacent to our out-of-sample mentalizing ROI, being just slightly anterior and superior to the region of precuneus identified by Neurosynth. We extracted mean parameter estimates from the region identified by the whole-brain analyses to confirm that outliers were not responsible for this pattern. We also ran the comparable linear model using mean parameter estimates from the original Neurosynth-derived precuneus ROI, which were highly correlated with parameter estimates from the post hoc identified region of the precuneus (*p* < 0.001), and showed the same pattern. For completeness, we then ran linear models with all a priori ROIs and related single waves (both wave 1 and 2) of brain activity to the IRI-PT difference scores reflecting change over time in perspective-taking tendency, but there were no other significant associations.

## 4 Discussion

In this study, we examined the development of, and individual differences in, perspective-taking tendencies and activation of mentalizing brain regions during social self-evaluation in adolescent girls. We also explored how these processes relate to each other. We found that self-reported perspective-taking tendencies increased with age on average across early-to-middle adolescence, and observed substantial individual variability in IRI-PT scores. In addition, our BLCS model showed that perspective-taking tendency at wave 1 negatively predicted latent change in perspective-taking tendency across both waves. Similarly, activity in mentalizing regions during social self-evaluation at wave 1 in our BLCS model negatively predicted latent change in brain activity across both waves. We also observed that dmPFC activity during social self-evaluation did not decrease with age. These findings build on the small number of existing longitudinal studies showing an increase in perspective-taking tendencies with age among adolescents and converge with more recent longitudinal findings that do not show decreases in dmPFC activity across adolescence. Our study holds important implications for positive and successful social development among adolescent girls.

### 4.1 Self-reported perspective-taking tendencies increase with age

The developmental increase in self-reported perspective-taking tendencies seen in our study aligns with previous research suggesting a positive association between perspective-taking tendencies and age across adolescence (Davis and Franzoi, 1991, Van der Graaff et al., 2014). In addition, our BLCS model indicates that lower perspective-taking tendency at wave 1 predicts greater latent change in perspective-taking tendency across the two waves. This may reflect the impact of the timing of perspective-taking development. For instance, an individual with lower perspective-taking tendency at wave 1 may subsequently experience greater developmental change in perspective-taking, whereas those with greater perspective-taking tendency at that time may have already experienced a portion of that change prior to wave 1 and thus have less change between waves. Our longitudinal work is an important contribution as cross-sectional research adolescent perspective-taking has generated incongruent findings (Hawk et al., 2013, Karniol et al., 1998) and limited longitudinal research on this topic is currently available. Our sample of adolescent girls extends existing longitudinal work on self-reported perspective-taking tendencies, which has typically focused on middle-to-late adolescence (Van der Graaff et al., 2014), by also incorporating early adolescence. Finally, we observe substantial individual variability in self-reported perspective-taking tendencies which aligns with similar previously reported findings (Farrell and Vaillancourt, 2021).

### 4.2 The relationship between mentalizing ROI activity during social self-evaluation and age

While the positive relationship between dmPFC activity and age (β = 0.120, *p* = 0.045) did not survive correction for multiple comparisons, this finding differs from previously published accounts suggesting that mPFC activity, particularly dmPFC activity, during mentalizing peaks in early adolescence or decreases across adolescence (Andrews et al., 2021, Blakemore, 2008, Grossmann, 2013, Johnson et al., 2009). In contrast, our finding may be more aligned with research showing an increase in dmPFC activity in early-to-middle adolescence. Indeed, recent longitudinal work has actually shown a quadratic relationship of dmPFC activity with age across adolescence. For example, one study of 34 adolescents found a quadratic trajectory of dmPFC activity across early-to-mid adolescence using three waves of data (van Buuren et al., 2022). This finding is bolstered by a study of 189 participants, where the oldest participants were only 24 years old, that reported a quadratic trend of self-evaluative dmPFC activity across adolescence with a very slight decrease beginning in early adulthood (Van Der Cruijsen et al., 2023). In line with recent findings, our work suggests that the mPFC is still recruited across adolescence during social cognitive tasks and its trajectory of functional development may be more nuanced than previously suggested.

Additionally, our BLCS model (Fig. 5) showed that lower average activity at wave 1 in these regions significantly predicted greater latent change in activity in these brain regions. This finding aligns with the idea that an adolescent who is further “behind” in their developmental trajectory of mentalizing neural activation, may be more likely to see greater subsequent change in mentalizing neural activity compared to an adolescent who is at a more advanced stage of mentalizing neural activity by wave 1 (see Beck et al., 2023 for similar findings whereby there is a significant, negative path from wave 1 of a given variable and that same variable’s latent change).

### 4.3 The relationship between self-reported perspective-taking tendency and activity in mentalizing brain regions during self-evaluation

We did not find significant cross-domain paths between perspective-taking tendencies and ROI activation in our BLCS model. In other words, paths relating perspective-taking tendencies at wave 1 to the mentalizing neural variable at wave 2 or latent change in the latent mentalizing neural variable between waves were not significant (i.e., latent mentalizing neural activity during wave 1 did not significantly predict perspective-taking tendencies scores at wave 2 or latent change in IRI-PT scores).

We also conducted a series of exploratory, post hoc analyses that generated limited findings of interest. However, greater increase in perspective-taking tendencies across the two waves was associated with more activity in the precuneus during self > change at the second wave, both for our Neurosynth-derived precuneus ROI and an adjacent cluster identified through a whole-brain, voxel-wise search (see Supplementary Figure S7).

### 4.4 The relationship between self-reported perspective-taking tendencies and social self-evaluation

Adolescent girls in our study who believed they had a higher tendency to take others’ perspectives also tended to view themselves as more prosocial and less antisocial. These findings align with research suggesting that adolescent perspective-taking tendencies and abilities are associated with, or result in, increased prosociality—although some longitudinal work has found only indirect effects of this relationship which was mediated by more affective components such as empathic concern (Gülseven et al., 2020, Güroğlu et al., 2014, Tamnes et al., 2018, Van der Graaff et al., 2018). Existing work suggests cognitive empathy, perspective-taking, or Theory of Mind tendency or ability is negatively associated with antisociality, although these findings are mixed and null results have also been reported (Anastassiou-Hadjicharalambous and Warden, 2008, Anderson et al., 2024, Zafirakis, 2009). While the relationship between these multidimensional constructs may appear robust at first blush, it is nuanced and may depend on measurement and context—driving researchers to call for the precise conceptualization and measurement of these constructs (Guazzelli Williamson and Mills, 2023, Hodges and Wise, 2016, Sassenrath et al., 2022, Silke et al., 2018). We highlight that we extend existing work by focusing on these relationships through the lens of self-perception, specifically: our results indicate that adolescent girls’ beliefs about their perspective-taking tendency relate to their self-evaluated prosociality and antisociality.

### 4.5 Limitations and future directions

Our study is not without limitations. As our sample was composed of youth assigned female at birth, our results may not be safely extrapolated to male adolescents. Most work investigating sex and gender differences in mentalizing and perspective-taking has been conducted among adults, and recent studies with large samples have found minimal or no differences in behavioral results between men and women (Baez et al., 2017, McDonald and Kanske, 2023). Even less work has probed these relationships among adolescents and those that do show mixed findings. One challenge of parsing the existing literature includes the conflation of sex and gender and so we recommend addressing sex and gender separately going forward. Some cross-sectional gender (Smith et al., 2016) and sex (Hawk et al., 2013) differences have been reported in IRI-PT scores among adolescents. Some theoretical accounts and longitudinal studies also suggest that developmental trajectories of mentalizing and perspective-taking tendencies may differ between adolescent boys and girls, with the prevailing theory that girls develop social cognitive capacities earlier than boys (Barr and Higgins-D’Alessandro, 2007, Hollarek and Lee, 2022, Smith et al., 2016, Van der Graaff et al., 2014). However, similar to recent studies among adults, much of the research on adolescents has reported small effect sizes or no differences in mentalizing or perspective-taking tendencies between boys and girls (Hollarek and Lee, 2022, Trentini et al., 2022, Van der Graaff et al., 2014). Additionally, while we chose not to analyze puberty because existing work on mentalizing has largely focused on age (not puberty), we note that this could be a promising area for future research, and may even help explain incongruous findings related to sex differences. Moreover, sex differences in the timing of puberty onset—and the subsequent onset of adolescence and brain development changes—may also limit the generalizability of the current study beyond girls.

Additionally, with a sample of our size, there is some question as to whether we were appropriately powered to detect effects in our analyses of Hypothesis 3. Power to detect effects can also be influenced by the magnitude of neuroimaging contrast. No doubt, our sample of 174 participants is 10-fold larger than some of the previous studies investigating activation of mentalizing regions during self-evaluation among adolescents and thus an important extension of this work (Jankowski et al., 2014, Pfeifer et al., 2009). Future research might employ methods to increase power and sensitivity to detect developmental trajectories by pooling information across brain regions (Cosme et al., 2022).

The measures used to capture the constructs of interest in this study also have limitations. For example, the IRI-PT is a self-report measure that purportedly captures one’s tendency to perspective-take but may not be safely extrapolated to mentalizing ability or degree (Guazzelli Williamson and Mills, 2023). In other words, while IRI-PT may capture one’s belief about their propensity to think about another person’s point of view, it is distinct from one’s accuracy when thinking of another individual’s perspective or underlying mental states. Additionally, there is evidence that IRI-PT scores may be influenced by social desirability bias (Constantine, 2000, Laurent and Hodges, 2009). The fact that self-report measures were significantly correlated with each other (e.g., IRI-PT and self-reported trait adjective endorsement), but not with most indices of brain activity may be a reflection of such self-report biases, as ratings of prosocial self-concept may also be subject to social desirability bias. Finally, it is not possible to determine with certainty what activity in the pre-specified mentalizing ROIs during our social self-evaluation task reflects. For instance, it is not possible to conclude that BOLD signal in these regions reflects mentalizing activity or evaluation of a looking-glass self.

Future studies will be well-served in conducting longitudinal follow-ups with additional waves of data to understand how perspective-taking tendencies and mentalizing during social self-evaluation continue to develop during adolescence. Studies that cover a larger age range may be better suited to capture development trajectories and relations between these processes. Our intent for this paper was to utilize a recently described analytical strategy (BLCS modeling; Kievit et al., 2018) that by definition constrained us to using two waves of data. However, models with three or more waves of data allow for the possibility of fitting individual participant slopes with a lower risk of overfitting, more reliable estimation of an overall growth trend, and separation of between- versus within-person effects (Parsons and McCormick, 2024). Such a strategy would help account for individual differences in rate of development which is important as adolescents may undergo unique developmental trajectories (as has been seen in individual variability of structural brain development; Mills et al., 2021).

Future studies may also wish to examine whether results differ when using a voxel-wise whole brain “signature” of mentalizing rather than collapsing across voxels in prespecified as ROIs as was done in the present study. Because this approach uses patterns of activation across the whole brain to develop predictive models, neural signatures may be more sensitive and specific indicators of a given psychological process than ROIs (Chang et al., 2015, Woo et al., 2017). Furthermore, this increased sensitivity and specificity can allow neural signatures to measure spontaneous, uninstructed engagement in psychological processes, such as mentalizing, which may enable the use of more ecologically valid tasks (Cosme et al., 2020). In addition, a behavioral measure of mentalizing ability or capacity may be useful in place of, or in addition to, the IRI-PT as it may more closely fit with activity in mentalizing brain regions, thus bolstering our confidence that such neural activity truly reflects mentalizing processes.

## 5 Conclusion

The development of perspective-taking and a stable, multifaceted self-concept during adolescence are associated with positive functional outcomes, ranging from academic and occupational success to social well-being (Crone et al., 2022). Indeed, the successful development of these capacities allows adolescents to build strong relationships, and become effective, contributing members of our respective communities (Crone et al., 2022, Crone and Fuligni, 2020, Hollarek and Lee, 2022). Our study offers an important step towards understanding how perspective-taking tendency and mentalizing processes relate to each other, change over time, and vary at an individual level among adolescent girls during early to middle adolescence. Our findings suggest that self-reported perspective-taking tendencies among adolescent girls tend to increase with age and are associated with increased self-evaluated prosociality and decreased self-evaluated antisociality. Notably, after correction for multiple comparisons, activity in our mentalizing ROIs (dmPFC, vmPFC, TPJ, and precuneus) during social self-evaluation did not significantly change with age, which contrasts with longer-standing theories and reports that activity of mentalizing brain regions, such as dmPFC, decrease across adolescence. While cross-domain paths of our BLCS model were not significant, post hoc analyses demonstrated that, on average, individuals who displayed greater increases in perspective-taking tendency across both waves also had greater BOLD activity in portions of the precuneus at wave 2. Moreover, there appears to be substantial individual variability in perspective-taking tendencies and their relation to age and brain activity across waves—a topic that warrants further research. Additional longitudinal research contextualizing brain-behavior interactions of perspective-taking tendencies—both within and beyond social self-evaluative contexts—across development could further illuminate the emergence and progression of these processes throughout adolescence.

## Funding

This work is supported in part by awards R01/R56 MH107418 and R01 MH127408. VGW is supported by a Ford Foundation Predoctoral Fellowship and has been previously supported by the National Institute of Mental Health (F31 MH130138).

## CRediT authorship contribution statement

**Flournoy John C.:** Writing – review & editing, Methodology, Investigation, Formal analysis, Conceptualization. **Cheng Theresa W.:** Writing – review & editing, Supervision, Investigation. **Cosme Danielle:** Writing – review & editing. **Byrne Michelle L.:** Writing – review & editing, Investigation. **Allen Nicholas B.:** Writing – review & editing. **Pfeifer Jennifer H.:** Writing – review & editing, Writing – original draft, Supervision, Software, Resources, Project administration, Methodology, Investigation, Conceptualization. **Guazzelli Williamson Victoria:** Writing – review & editing, Writing – original draft, Visualization, Validation, Project administration, Methodology, Investigation, Funding acquisition, Formal analysis, Data curation, Conceptualization. **Barendse Marjolein E. A.:** Writing – review & editing, Validation, Supervision, Methodology, Investigation, Formal analysis. **Chavez Samantha J.:** Writing – review & editing, Validation, Supervision, Methodology, Investigation, Formal analysis, Data curation.

## Declaration of Competing Interest

The authors declare the following financial interests/personal relationships which may be considered as potential competing interests: Victoria Guazzelli Williamson reports financial support was provided by Ford Foundation Predoctoral Fellowship and by the National Institute of Mental Health (F31 MH130138). Jennifer H Pfeifer reports financial support was provided by National Institute of Mental Health (Grants R01/R56 MH107418 and R01 MH127408). If there are other authors, they declare that they have no known competing financial interests or personal relationships that could have appeared to influence the work reported in this paper.

## Footnotes

1 At study enrollment, 171 identified as female and 3 as non-binary
