# Supplementary material for: A longitudinal neuroimaging study of adolescent girls’ mentalizing and perspective-taking tendencies
Source: Dev Cogn Neurosci. 2025 Feb 7;72:101526. doi: 10.1016/j.dcn.2025.101526 (PMC11891602; doi:10.1016/j.dcn.2025.101526)
Supplement: Supplementary file 1 — Supplementary material [file mmc1.docx]

**Supplementary Materials**

Supplementary Figure S1. IRI-PT Score Distribution.


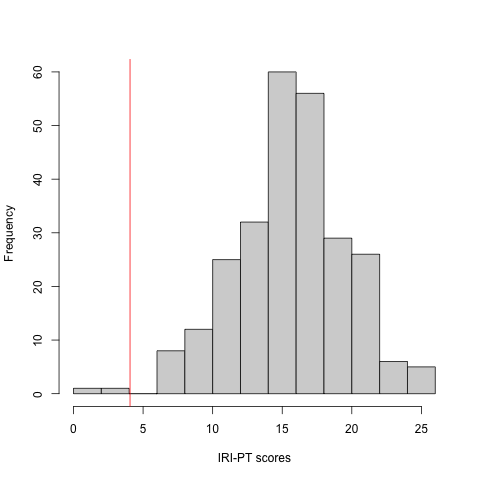

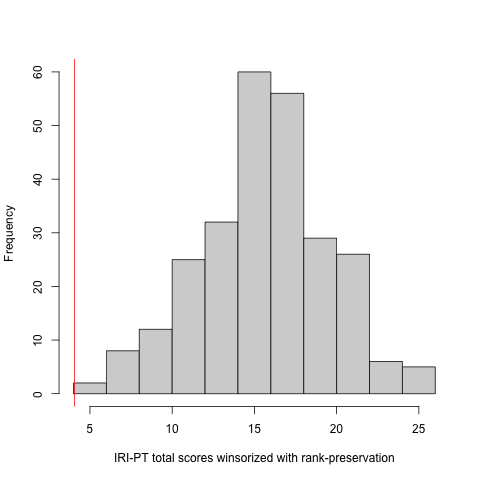


Supplementary Figure S1 Legend. IRI-PT score distributions before winsorizing and after winsorizing with rank order preservation. Values to the left of the red line represent outliers.

Supplementary Figure S2. IRI and Self-Evaluation Correlation Matrix


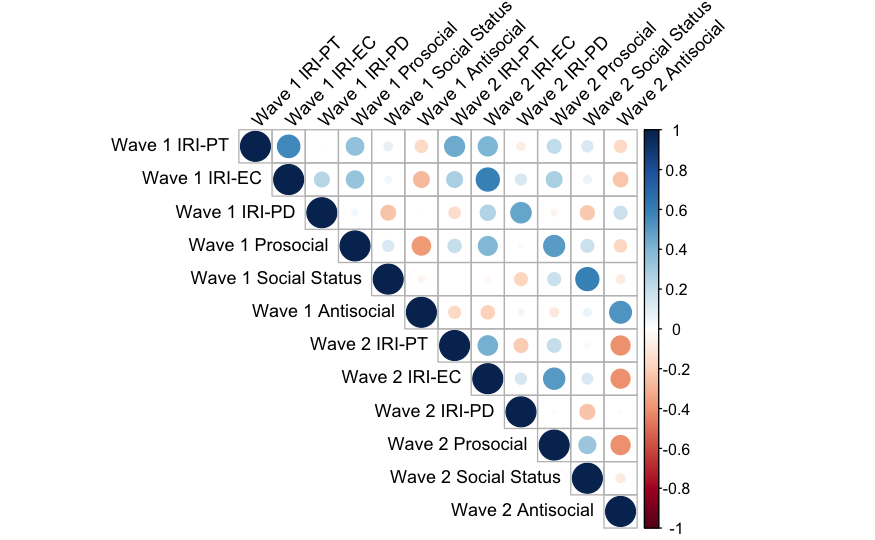


Supplementary Figure S2 Legend. This correlation matrix includes proportions of endorsement for each adjective type (e.g., prosocial, social status, and antisocial), and scores from three of the four IRI subscales as specified in the preregistration including the perspective-taking subscale (IRI-PT), as well as the personal distress subscale (IRI-PD), and empathic concern subscale (IRI-EC).

Supplementary Figure S3. ROI Correlation Matrix

​​
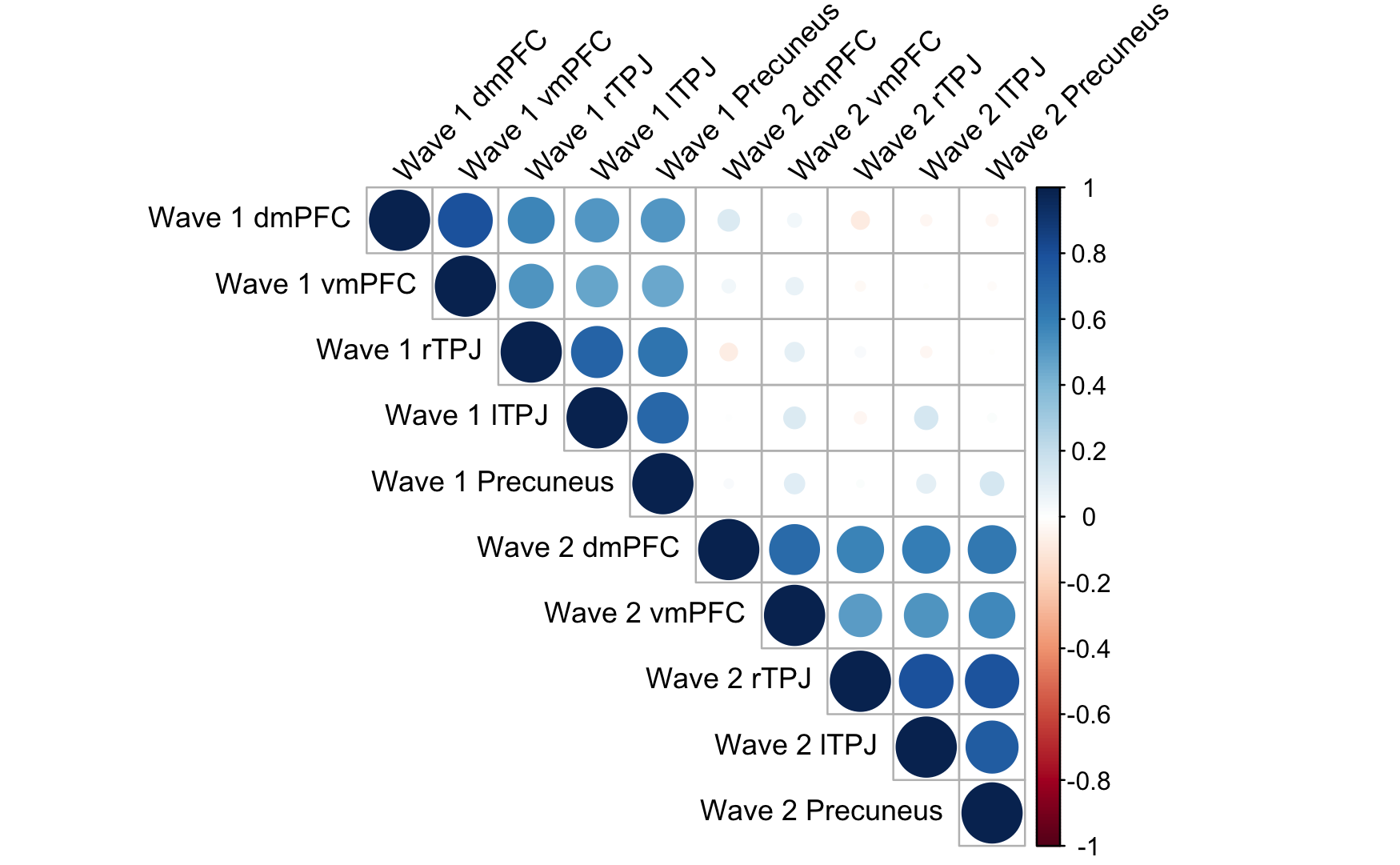


Supplementary Figure S3 Legend. Correlation matrix relating activity at waves 1 and 2 in dorsomedial prefrontal cortex (dmPFC), ventromedial prefrontal cortex (vmPFC), right temporoparietal junction (rTPJ), left temporoparietal junction (lTPJ), and precuneus.

Supplementary Figure S4. Association Between ROI and Age


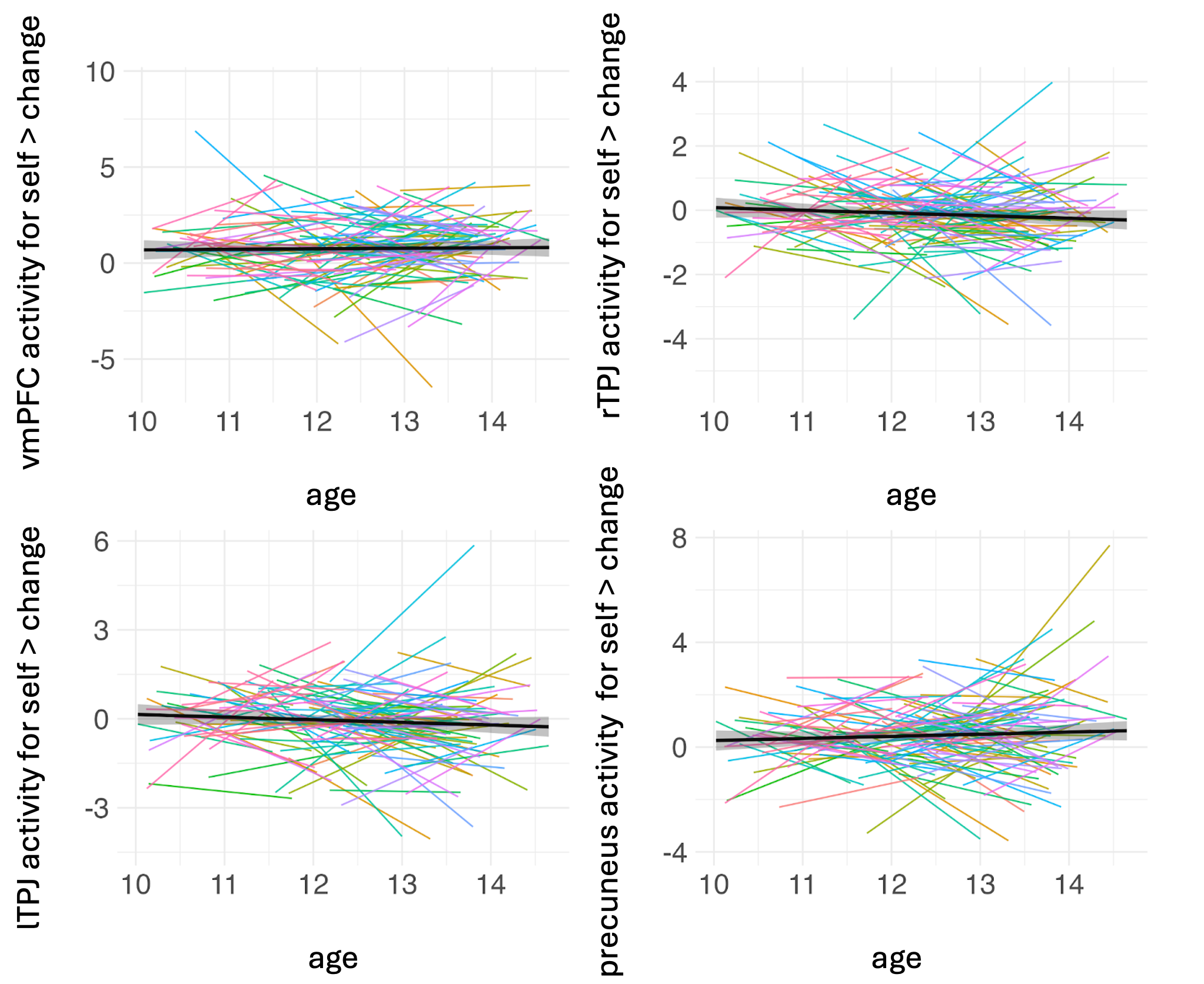


Supplementary Figure S4 Legend. Panel showing the association between age and activity in ventral medial prefrontal cortex (vmPFC), right temporal parietal junction (rTPJ), left temporal parietal junction (lTPJ) and precuneus. The black line represents the predicted values based on the mixed-effects model, whereas the colored lines represent raw (not model-predicted) individual trajectories. The 95% confidence interval is depicted via a gray band.

Supplementary Figure S5. Association Between IRI-PT Score and ROI


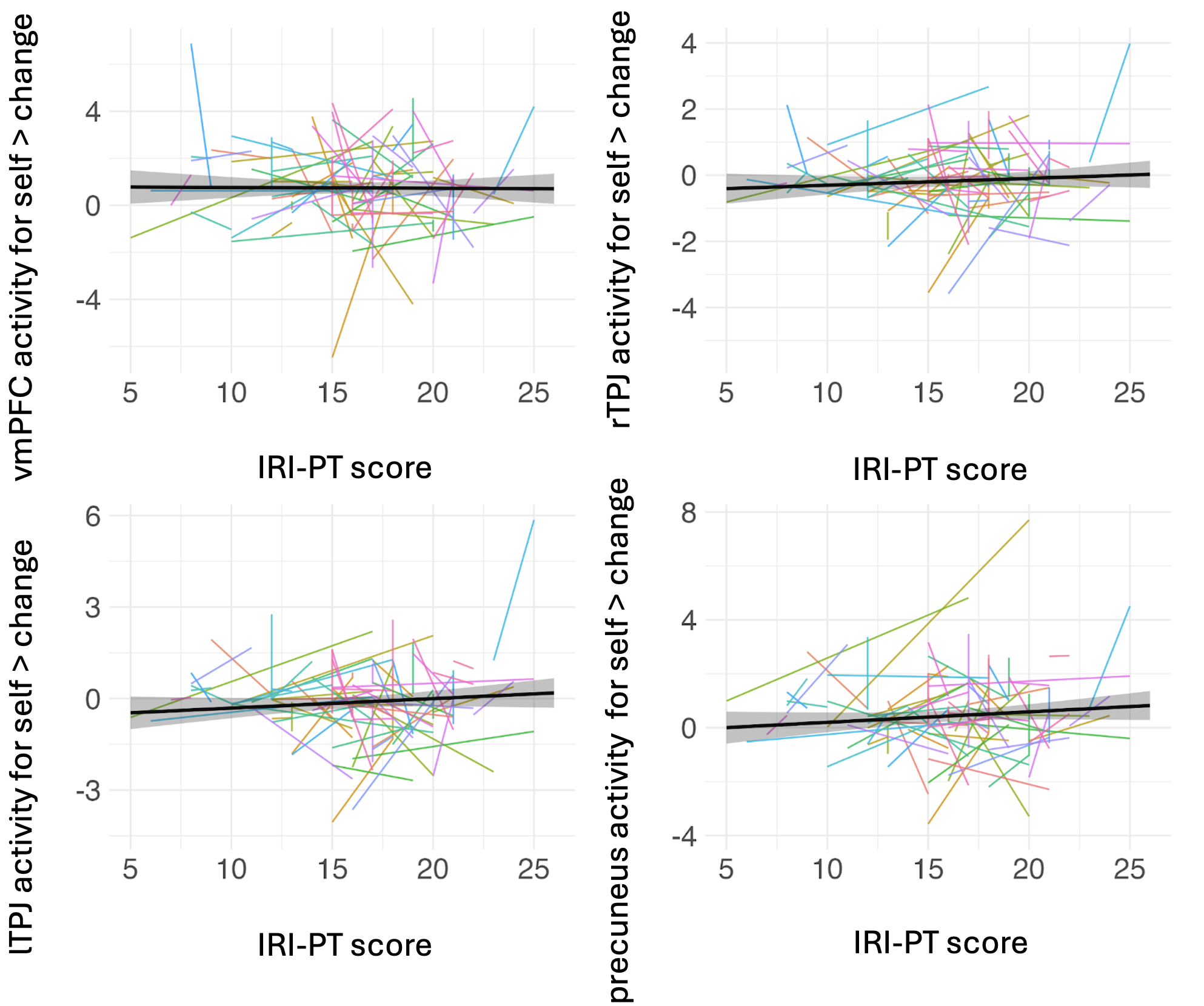


Supplementary Figure S5 Legend. Panel showing the association between IRI-PT score and activity in ventral medial prefrontal cortex (vmPFC), right temporal parietal junction (rTPJ), left temporal parietal junction (lTPJ) and precuneus. The black line represents the predicted values based on the mixed-effects model, whereas the colored lines represent raw (not model-predicted) individual trajectories. The 95% confidence interval is depicted via a gray band.

Supplementary Analysis 1.

To address the original final hypothesis from our preregistration (Hypothesis 4 in this manuscript), we originally attempted to fit a Bivariate Latent Change Score (BLCS) model ([Kievit et al., 2018)](https://www.zotero.org/google-docs/?R3cOJN) of perspective-taking tendency and activity in our mentalizing ROIs during social self-evaluation using a latent neural variable. Given the theory that mentalizing and perspective-taking are likely complex computational processes involving a network of regions working in conjunction—instead of being based on activity within any one region on its own [(Blakemore, 2008; Blakemore & Mills, 2014)](https://www.zotero.org/google-docs/?1b2ZVs)—we first used Confirmatory Factory Analysis to attempt to create a latent variable model of mentalizing neural activity that would capture the shared variance in these ROIs to be used as our measure of mentalizing neural activity. We were ultimately able to create a latent variable of mentalizing that met our goodness of fit criteria and was subsequently used in the BLCS model. This latent variable was modeled with one latent factor for each wave, with ROIs serving as indicators. Factor loadings for the ROIs were constrained to be equal across waves 1 and 2, ensuring consistent measurement of the mentalizing construct over time. Within each wave, ROIs were allowed to covary, and these covariance parameters were constrained to be equal across waves. However, results from our Confirmatory Factor Analysis (CFA) of the latent mentalizing neural variable and the BLCS with the latent mentalizing neural variable raised concerns, generating a number of warnings including that the variance-covariance matrix of the estimated parameters was not positive definite which may indicate that the model is not identified, as well as warnings about negative variances (i.e., Heywood cases), suggesting that the model may be mis-specified [(Kolenikov & Bollen, 2012)](https://www.zotero.org/google-docs/?6kDZtl). Thus, we will report output from this model on our OSF repository (https://osf.io/xmb9w/) but do not interpret the model findings in text.

We also ran a BLCS model with the aforementioned latent variable where covariances were not constrained to be equal across waves. The same paths of interest were significant in this model as in the other BLCS models (e.g., IRI-PT at wave 1 predicted change in IRI-PT and neural activity at wave 1 predicted change in neural activity; see Figure 5) but this model displayed worse fit and thus its statistical findings are not reported in the main text. In addition, removing latent change from the BLCS model did not significantly worsen the overall fit of the model, suggesting that model instability is largely due to the latent mentalizing neural variable, and not aspects of the BLCS such as latent change.

We also conducted simple linear regressions of IRI-PT and individual ROI change scores with age as a covariate in the model (ΔROI ~ ΔIRI-PT + age_w1) as these were preregistered contingency plans should the BLCS model fail. ΔROI was calculated via ROIw2 - ROIw1 and ΔIRI-PT was calculated via IRI-PTw2 - IRI-PTw1. No IRI-PT change scores were significantly associated with ROI change scores. Of note, the precuneus had the largest effect size, although this relationship was ultimately not significant (β = 0.11, *SE* = 0.05, *t* = 983, *p* = 0.051).

Finally, we preregistered running a BLCS model with activity from the auditory cortex (ROIs and other model output will be available in our Open Science Framework repository at https://osf.io/xmb9w/). Similar to the model of mean ROI activity reported in text (Figure 5), we averaged across two auditory ROIs to generate a neural indicator of activity for each wave. This just-identified model differed from our findings related to activity in mentalizing ROIs (e.g., Figure 5) in that none of the primary paths related to the auditory ROIs were significant, including from activity in the auditory cortex at wave 1 to latent change in the auditory cortex across both waves (see Supplementary Figure S6).

Supplementary Figure S6: Bivariate Latent Change Score (BLCS) Model with Auditory Cortex ROI


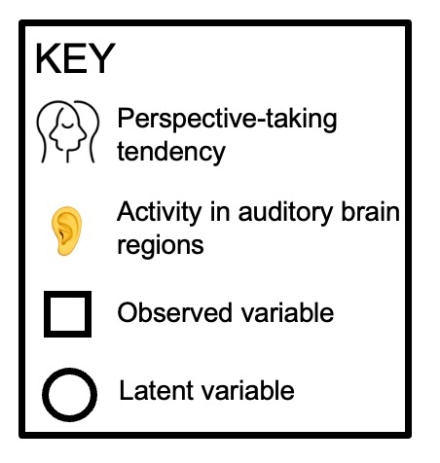

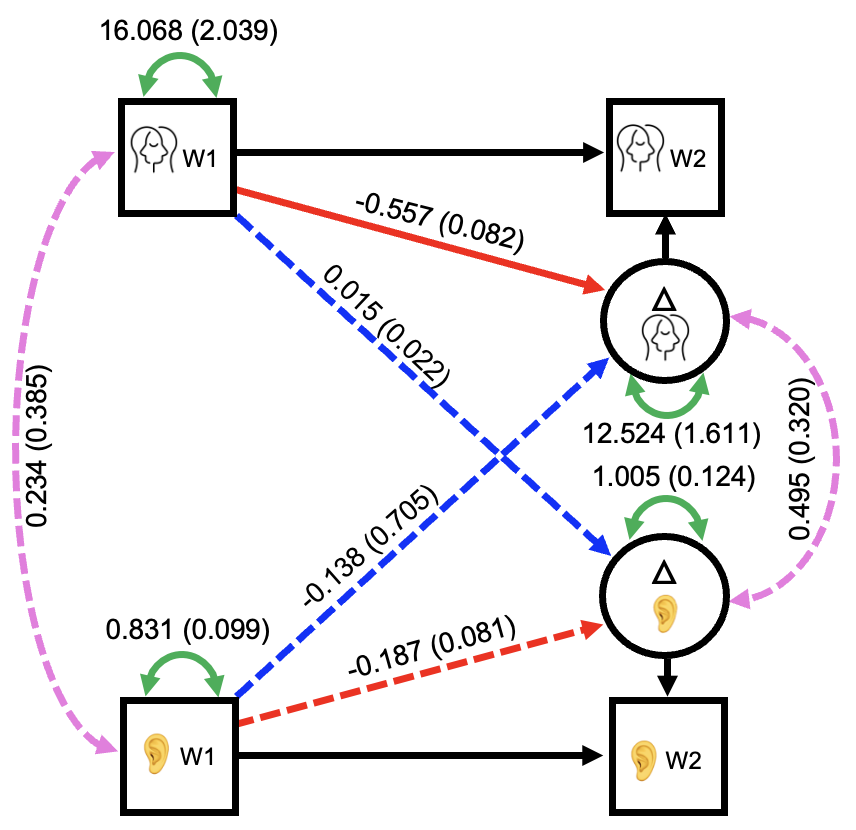


Supplementary Figure S6 Legend. This BLCS model shows significant paths indicated via solid lines and non-significant paths are represented via dashed lines. Estimates for each path are presented, with standard errors listed in parentheses. Latent variables are indicated via circles (e.g., latent change scores) whereas observed variables are depicted via squares.

Supplementary Analysis 2.

In line with our preregistered addendum (<https://osf.io/b4qus>), we similarly attempted to compute a latent IRI-PT variable comprised of the 7 IRI-PT items. Similar to the latent neural variable of mentalizing, the model that met our preregistered goodness of fit criteria had one latent factor per wave, each indicated by an individual IRI-PT item, which were estimated freely, constrained across waves, and allowed to correlate within waves. However, as with the latent variable of mentalizing brain activity, this latent variable generated similar warning messages and negative variances. We were ultimately unable to extract factor scores from the latent IRI-PT model.

Post-hoc analyses: Relating ROI activation to self-evaluation task responses

Supplementary Table S1. Multilevel models associating endorsement of each adjective type to mean ROI activity

|  | **β (95% CI)** | ***SE*** | ***df*** | ***t*** | ***p*** | ***SD* of random effect** |
| --- | --- | --- | --- | --- | --- | --- |
| Prosocial ~ ROIs | -0.04 (-0.15, 0.07) | 0.00 | 218.11 | -0.65 | .519 | 0.07 |
| Social Status ~ ROIs | -0.01 (-0.09, 0.11) | 0.01 | 220.67 | 0.16 | .872 | 0.16 |
| Antisocial ~ ROIs | 0.01 (-0.09, 0.12) | 0.01 | 228.33 | 0.25 | .803 | 0.12 |

Supplementary Table S2. Multilevel models associating endorsement of each adjective type to dmPFC activity

|  | **β (95% CI)** | ***SE*** | ***df*** | ***t*** | ***p*** | ***SD* of random effect** |
| --- | --- | --- | --- | --- | --- | --- |
| Prosocial ~ dmPFC | -0.06 (-0.17, 0.05) | 0.00 | 217.00 | -1.13 | .261 | 0.07 |
| Social Status ~ dmPFC | 0.03 (-0.08, 0.13) | 0.01 | 219.46 | 0.52 | .603 | 0.16 |
| Antisocial ~ dmPFC | 0.02 (-0.09, 0.13) | 0.01 | 226.97 | 0.38 | .706 | 0.13 |

Supplementary Table S3. Multilevel models associating endorsement of each adjective type to vmPFC activity

|  | **β (95% CI)** | ***SE*** | ***df*** | ***t*** | ***p*** | ***SD* of random effect** |
| --- | --- | --- | --- | --- | --- | --- |
| Prosocial ~ vmPFC | -0.04 (-0.15, 0.07) | 0.00 | 215.68 | -0.79 | .432 | 0.07 |
| Social Status ~ vmPFC | 0.03 (-0.07, 0.14) | 0.01 | 219.33 | 0.62 | .535 | 0.16 |
| Antisocial ~ vmPFC | 0.02 (-0.09, 0.13) | 0.01 | 226.62 | 0.404 | .687 | 0.13 |

Supplementary Table S4. Multilevel models associating endorsement of each adjective type to rTPJ activity

|  | **β (95% CI)** | ***SE*** | ***df*** | ***t*** | ***p*** | ***SD* of random effect** |
| --- | --- | --- | --- | --- | --- | --- |
| Prosocial ~ rTPJ | 0.02 (-0.09, 0.13) | 0.00 | 225.56 | 0.33 | .742 | 0.08 |
| Social Status ~ rTPJ | -0.01 (-0.12, 0.09) | 0.01 | 228.31 | -0.22 | .825 | 0.16 |
| Antisocial ~ rTPJ | -0.00 (-0.11, 0.11) | 0.01 | 235.88 | -0.07 | .946 | 0.13 |

Supplementary Table S5. Multilevel models associating endorsement of each adjective type to lTPJ activity

|  | **β (95% CI)** | ***SE*** | ***df*** | ***t*** | ***p*** | ***SD* of random effect** |
| --- | --- | --- | --- | --- | --- | --- |
| Prosocial ~ lTPJ | -0.01 (-0.12, 0.11) | 0.00 | 237.57 | -0.13 | .894 | 0.07 |
| Social Status ~ lTPJ | -0.03 (-0.13, 0.08) | 0.01 | 236.07 | -0.49 | .623 | 0.16 |
| Antisocial ~ lTPJ | -0.01 (-0.12, 0.10) | 0.01 | 244.73 | -0.22 | .825 | 0.13 |

Supplementary Table S6. Multilevel models associating endorsement of each adjective type to precuneus activity

|  | **β (95% CI)** | ***SE*** | ***df*** | ***t*** | ***p*** | ***SD* of random effect** |
| --- | --- | --- | --- | --- | --- | --- |
| Prosocial ~ precuneus | -0.03 (-0.15, 0.08) | 0.00 | 242.57 | -0.60 | .549 | 0.07 |
| Social Status ~ precuneus | -0.01 (-0.11, 0.10) | 0.01 | 240.29 | -0.10 | .920 | 0.16 |
| Antisocial ~ precuneus | 0.02 (-0.09, 0.13) | 0.01 | 248.18 | 0.37 | .709 | 0.13 |

Post-hoc analyses: Whole-brain search for neural regions associated with concurrent perspective-taking tendencies and change in perspective-taking tendencies across waves

Supplementary Table S7. Peak activity from whole brain models run in SPM12

|  | **Positive Correlation** | | | | **Negative Correlation** | | | |
| --- | --- | --- | --- | --- | --- | --- | --- | --- |
| **Model** | **Coordinates** | ***k_E_*** | ***t*** | ***p*** | **Coordinates** | ***k_E_*** | ***t*** | ***p*** |
| Wave 1 self > baseline and IRI-PT | 10, -74, 8  -2, -88, 10 | 275  192 | 4.34  3.75 | 0.000  0.000 | NA | NA | NA | NA |
| Wave 1 self > baseline and IRI-PT difference score | NA | NA\| | NA | NA | -14, -24, -2 | 78 | 4.65 | 0.000 |
| Wave 1 change > baseline and IRI-PT | 16, -70, 10  18, -92, 24 | 2339  86 | 5.23  4.39 | 0.000  0.000 | NA | NA | NA | NA |
| Wave 1 change > baseline and IRI-PT difference scores | 32, 44, 42 | 102 | 4.49 | 0.000 | NA | NA | NA | NA |
| Wave 1 self > change and IRI-PT | NA | NA | NA | NA | NA | NA | NA | NA |
| Wave 1 self > change and IRI-PT difference scores | NA | NA | NA | NA | NA | NA | NA | NA |
| Wave 2 self > baseline and IRI-PT | 22, -72, 52  -46, -40, 50 | 218  113 | 4.50  3.73 | 0.000  0.000 | NA | NA | NA | NA |
| Wave 2 self > baseline and IRI-PT difference scores | NA | NA | NA | NA | NA | NA | NA | NA |
| Wave 2 change > baseline and IRI-PT | NA | NA | NA | NA | 46, -52, 18 | 139 | 4.26 | 0.000 |
| Wave 2 change > baseline and IRI-PT difference scores | NA | NA | NA | NA | -30, -52, -4 | 106 | 4.05 | 0.000 |
| Wave 2 self > change and IRI-PT | 22, -72, 50  8, -64, 62  -30, -68, 26  -50, -58, -2  -36, -34, 52  -20, -64, 54  50, -46, -16 | 185  313  82  86  614  230  79 | 4.72  4.27  4.16  4.11  4.04  3.97  3.97 | 0.000  0.000  0.000  0.000  0.000  0.000  0.000 | NA | NA | NA | NA |
| Wave 2 self > change and IRI-PT difference scores | -2, -58, 48 | 114 | 4.07 | 0.000 | NA | NA | NA | NA |

Supplementary Table S7 Legend: We used the joint magnitude-extent threshold of .001 uncorrected, 66 voxels as determined by Barendse, Cosme, et al., 2020. T-values (*t*) and p-values (*p*) of peak voxel activity are reported along with the number of voxels in each cluster (*k_E_*).

Supplementary Figure S7: Positive correlation between change in IRI-PT score from wave 1 to wave 2 and BOLD signal during self > change at wave 2


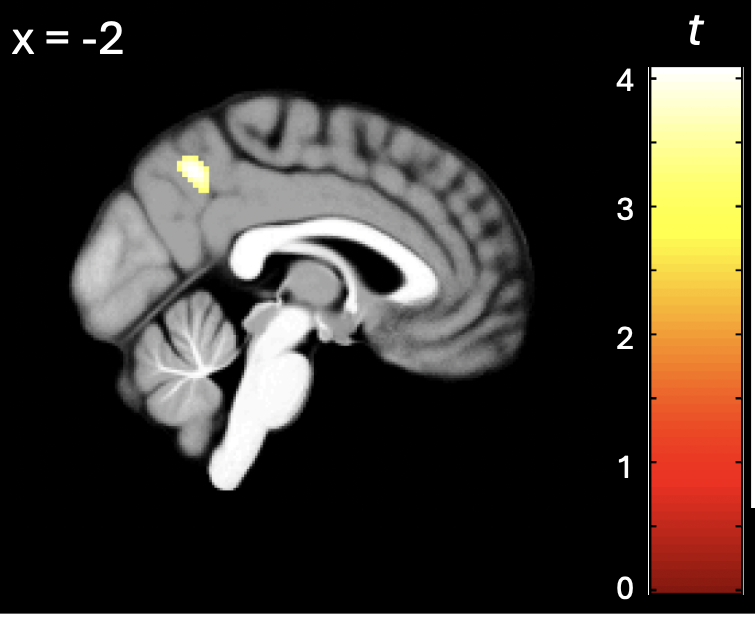


Supplementary Figure S7 Legend. This figure shows greater activity in a portion of the precuneus in the “self” compared to “change” conditions at wave 2. This cluster was identified through a whole-brain search for regions associated with the change in IRI-PT score from wave 1 to wave 2. On average, individuals who show a greater increase in IRI-PT scores across the waves also show greater activity in this region.
